# A Nutritional-Toxicological Assessment of Antarctic Krill Oil *versus* Fish Oil Dietary Supplements

**DOI:** 10.3390/nu6093382

**Published:** 2014-08-28

**Authors:** Susan M. Bengtson Nash, Martin Schlabach, Peter D. Nichols

**Affiliations:** 1 Griffith University, Environmental Futures Research Institute, Nathan, QLD 4111, Australia; 2 The Norwegian Institute for Air Research (NILU), Kjeller 2027, Norway; E-Mail: msc@nilu.no; 3 CSIRO Food and Nutrition, Oceans and Atmosphere Flagships, GPO Box 1538, TAS 7000, Australia; E-Mail: Peter.Nichols@CSIRO.au

**Keywords:** Antarctic krill oil, dietary supplements, persistent organic pollutants, long-chain omega-3 polyunsaturated fatty acids

## Abstract

Fish oil dietary supplements and complementary medicines are pitched to play a role of increasing strategic importance in meeting daily requirements of essential nutrients, such as long-chain (≥C_20_, LC) omega-3 polyunsaturated fatty acids and vitamin D. Recently a new product category, derived from Antarctic krill, has been launched on the omega-3 nutriceutical market. Antarctic krill oil is marketed as demonstrating a greater ease of absorption due to higher phospholipid content, as being sourced through sustainable fisheries and being free of toxins and pollutants; however, limited data is available on the latter component. Persistent Organic Pollutants (POP) encompass a range of toxic, man-made contaminants that accumulate preferentially in marine ecosystems and in the lipid reserves of organisms. Extraction and concentration of fish oils therefore represents an inherent nutritional-toxicological conflict. This study aimed to provide the first quantitative comparison of the nutritional (EPA and DHA) *versus* the toxicological profiles of Antarctic krill oil products, relative to various fish oil categories available on the Australian market. Krill oil products were found to adhere closely to EPA and DHA manufacturer specifications and overall were ranked as containing intermediate levels of POP contaminants when compared to the other products analysed. Monitoring of the pollutant content of fish and krill oil products will become increasingly important with expanding regulatory specifications for chemical thresholds.

## 1. Introduction

Fish are a nutrient-dense food source. The role of marine-derived, long-chain (LC) (>C_20_) omega-3 (ω3) polyunsaturated fatty acids (LC-PUFA), in the promotion of health is well established. Since early observations that Greenland Eskimos who subsisted on large amounts of fish suffered low levels of cardiovascular disease related mortality, epidemiological and experimental evidence has confidently shown the protective role of sufficient ω3 LC-PUFA intake against cardiovascular disease and certain types of cancer, e.g., [1,2]. In particular docosahexaenoic acid (DHA, 22:6ω3) and eicosapentaenoic acid (EPA, 20:5ω3), each with distinct roles in disease prevention, have been credited for their contribution to a healthy diet. In addition to serving as energy stores, ω3 LC-PUFA form integral structural components of cellular membranes [3]. For example, ω3 LC-PUFA are highly concentrated in the cellular membranes of the retina and brain and accumulate there rapidly in the third trimester of foetal development. Gestational ω3 LC-PUFA restrictive studies have shown significant deleterious impact to off-spring visual acuity and cognitive function [4]. Finally, symptomatic alleviation with ω3 LC-PUFA intake has been reported for a broad range of health conditions. Anti-inflammatory properties of ω3 LC-PUFA provide a molecular basis for symptomatic alleviation of inflammatory disease such as rheumatoid arthritis, lupus and asthma [5,6,7]. More recently improvements in psychiatric disorders such as depression and schizophrenia with ω3 LC-PUFA administration have been observed [8].

Fish oil is also a rich source of lipid-soluble micronutrients such as vitamin D, which plays a fundamental role in bone health [9]. Consequently, The National Heart Foundation of Australia, in accordance with a host of international agencies, recommends consumption of fish at least twice a week. Paradoxically, modern diets in developed nations are characterised by severe ω3 LC-PUFA deficiency, reflecting low seafood intake. This was exemplified in a recent study which found 78% of the Australian population did not meet their daily recommended intake of ω3 LC-PUFA [10]. In fact, it must be considered that meeting health targets for seafood intake is not economically nor ecologically attainable for large fractions of the global population [11].

Effectively tackling dietary deficiency of seafood micronutrients would carry significant bearing on both the social and economic burden of disease. Increasing the dietary status of vitamin D alone in Western Europe has been estimated to alleviate the economic burden of disease by $293 billion per year [12]. In the absence of sufficient high quality, affordable seafood sources, dietary supplements and complementary medicines are pitched to play a role of increasing strategic importance.

A new product category has been launched on the omega-3 nutriceutical market and is currently gaining significant market share. A nutriceutical oil derived from Antarctic krill (*Euphausia superba*), a Euphausiid crustacean forming the basis of the Antarctic food web, has been marketed since 2002 and has recently become broadly available in Australia [13]. Marketing of krill oil centres on three characteristic properties of the oil. Krill oil contains the essential nutrient, choline and an antioxidant, astaxanthin. In addition, it is posed that Antarctic krill oil derived ω3 LC-PUFA is more bioavailable compared to fish oils. A higher fraction of ω3 LC-PUFA is associated with phospholipids in krill oil, compared to triacylglycerols in fish oils. This property has been theorised to improve absorption and bioavailability of ω3 LC-PUFA [14], based upon independent liposome carrier research [15]. Secondly, one major krill oil manufacturer has achieved Marine Stewardship Council certification of sustainability and, as a whole, the industry is often viewed as being sustainable due to the fact that the worldwide harvest constitutes only a minor fraction of established fishing quotas, e.g., [14,16]. It should be noted, however, that uncertainty surrounds the distribution and density of circumpolar krill stocks and therefore the robustness of fishery quotas remains debated [17]. Finally, krill oil is cited as being naturally free of toxins and pollutants [6]. Persistent Organic Pollutants (POPs) are toxic contaminants that bioaccumulate, and have been introduced to the environment since the mid-1900s. Their extreme persistence and effective environmental dispersal mechanisms have resulted in ubiquitous contamination of all environmental matrices. POPs are considered a substantial risk to human health [18] and are subject to the Stockholm Convention, a legally binding treaty signed by over 100 nations, and ratified by Australia in 2004 [19].

Within an ecosystem, lower trophic level species such as zooplankton, are often found to accumulate lower levels of POP contaminant burdens due to shorter life-spans. However, this cannot be assumed when comparing species from different ecosystems. Polar species are characterised by large body size and long life spans. Antarctic krill live to 5–7 years which is comparable to, or longer than, source species commonly used in fish oil production [13]. Similarly, Antarctic krill have demonstrated highly adaptable feeding, and in addition to their herbivorous feeding, have been observed to rely on cannibalism and detritivory to endure food deprivation [20]. This dietary flexibility also confounds their trophic placement and thus the POP bioaccumulation patterns of the species.

Historical or “legacy” POPs are chlorinated compounds. Their common molecular structures predict similar environmental behaviour. In the physical environment they are semi-volatile. Volatilized fractions will undergo progressive movement towards colder and colder climates experiencing “cold-trapping” at the poles of the earth [21]. In the particulate phase they will adhere strongly to organic matter representing an effective mechanism for transfer from the terrestrial to the aquatic environment and assimilation into food-webs. Consequently, the vast majority of human exposure to POPs occurs via seafood consumption [22,23]. This clear nutritional-toxicological conflict associated with seafood intake has urged the Codex Alimentarious Commission for Contaminants in Food to convene an expert consultancy on the risks and benefits of fish consumption [18]. In the case of fish oil dietary supplements, the scenario is even more acute. Legacy POPs are extremely lipophilic and accumulate in the fat reserves of animals. When the lipid fractions of seafood are selectively isolated and concentrated for administration as dietary supplements or complementary medicines, the seafood micronutrient:POP burden conflict is exacerbated. Indeed, repeated incidences of fish oil product recalls due to exceedance of POP safety guideline have occurred and are only likely to rise as the market expands and authorities pursue greater regulatory overview [24,25].

Recently we conducted the most comprehensive analytical survey of POPs in any Antarctic environmental matrix to date [26]. Our study centred on Antarctic krill, as POP vectors to the remainder of the Antarctic food-web, and extended across almost a quarter of the Antarctic continent. Our findings highlighted that Antarctic krill POP profiles were distinct from those typical of northern hemisphere species, but that they were not insubstantial. Indeed, for some compounds such as hexachlorobenzene (HCB), levels were comparable to or greater than those of similar trophic level species in other global regions. This work has prompted the following strategic examination of commercial krill oil products. Here we will assess krill oil POP burdens, as well as product nutritional lipid class and fatty acid profiles. These will be compared to those of other categories of commercial fish oil dietary supplements available on the Australian market.

## 2. Experimental Section

### 2.1. Product Selection

Four categories of seafood-oil dietary supplements were selected for analysis, namely, (i) krill oil; (ii) enriched (in terms of EPA + DHA) fish oil; (iii) nutriceutical formulations containing fish oil; and (iv) standard or budget grade 18:12 (EPA + DHA) fish oil (Table 1). Products representative of the two major krill oil manufacturers were selected under the krill oil category. For the other remaining categories, three representative and readily available brands were selected. Efforts were made to combine capsules from two separate batches of each product for each POP and FA analysis. This was achieved for all products except Blackmores Omega Liquid Fish Oil for which only a single batch number could be sourced. Full details of selected products and batch numbers are listed in Table 1.

### 2.2. Sample Analysis

#### 2.2.1. Lipid Extraction and Class Determination

Pre-weighed (*c.a*. 0.03 g) oil samples were used for lipid analyses. Individual capsule or liquid oil samples were cut open and dissolved in CHCl_3_. A known aliquot of total lipid (achieving a final concentration of approximately 10 mg lipid/mL CHCl_3_) was transferred into separate vials and made up to 1.5 mL of CHCl_3_.

#### 2.2.2. Fatty Acid (FA) Determination

An aliquot of the total lipid extract was trans-methylated by addition of MeOH/HCl/CHCl_3_ (3 mL 10:1:1, v/v/v, 80 °C/2h) to produce fatty acid methyl esters (FAME). After cooling the mixture and addition of 1 mL of water, FAME were extracted (3×) with 4:1 hexane/dichloromethane. A C19 FAME internal injection standard was added prior to analysis by gas chromatography (GC) using a GC (Agilent Technologies 7890A) equipped with a Supelco Equity™-1 fused silica capillary column (15 m × 0.1 mm internal diameter, 0.1 μm film thickness) [27]. GC-mass spectrometry (GC-MS) confirmed FAME identifications and was performed on a Finnigan Thermoquest GCQ GC-mass spectrometer fitted with a column of similar polarity to that described above, an on-column injector and using Thermoquest Xcalibur software (Austin, TX, USA). Helium was used as carrier gas and other operating conditions were as previously described [27]. The relative levels of individual FA were expressed as percent of total FA area. A catalogue of quantified FA is presented in Table 2. FA present at less than 0.5% of total FA in all products are grouped as Other FA; this FA group comprised 1.6%–4.2% of the total FA across the products analysed.

**Table 1:** Selected fish and krill oil nutriceutical products compared in this study. Capsules or subsamples from two separate batches of each product were pooled for each fatty acids (FA) analysis, except for Blackmores Omega Liquid Fish Oil for which only a single batch number could be sourced.

Krill Oil	Enriched Fish Oil	Formulations Containing Fish Oil	Standard 18:20 Grade Fish Oil
	BIO organics Super Liquid Fish Oil EPA 1.6 g; DHA 810 mg 5 mL serve (Batch 11448A and 11815A)	Blackmores Omega Joint “Mercury, PCB and dioxin tested” EPA 550 mg; DHA 120 mg 1000 mg capsule (Batch 252505 and 252076)	Nature’s Own Odourless Fish Oil 1000 mg EPA 180 mg; DHA 120 mg 1000 mg capsule (Batch 650566 and 652769)
Swisse Wild Krill Oil (NKO) EPA 47 mg; DHA 28 mg 333 mg capsule (Batch 022537 and 022538)	Blackmores Omega Liquid Fish Oil EPA 1.7g; DHA 1.1g 5 mL serve (Batch 10709101)	Nature’s Way Kids Smart EPA 28 mg; DHA 133 mg 1000 mg capsule (Batch B8514-1 and Batch C4401)	Blackmores Odourless Fish Oil 1000 mg EPA 180 mg; DHA 120 mg 1000 mg capsule (Batch 252461 and 253417)
Norkrill (Aker BioMarine) EPA 60 mg; DHA 28mg 500 mg capsule (Batch 390048 and 443003)	Bioceuticals OmegaSure Liquid Fish Oil EPA 1050 mg; DHA 750 mg 5 mL serve (Batch 26764 B L80 and 26764 C L80)	Blackmores Pregnancy and Breastfeeding Gold EPA 25 mg; DHA 125 mg 1000 mg serve (Batch 252025 and 250973)	Cenovis Fish oil 1000 mg EPA 180mg; DHA 120 mg 1000 mg capsule (Batch 650588 and 649319)

#### 2.2.3. Quality Control

For lipid class and FA profiling, commercial (Nuchek) and laboratory standards (e.g., tuna oil) of known composition were routinely analysed to both confirm component identifications and ensure data quality.

#### 2.2.4. Chemical Analysis

Oil samples were analysed for chlorobenzenes (hexa- and penta-chlorobenzene); chlorinated pesticides; hexachlorocyclohexanes (α-, β-, γ- HCH); the dichlorodiphenyltrichloroethane (DDT) group (o,p’-DDE, p,p’-DDE, o,p’-DDD, p,p’-DDD, o,p’-DDT, p,p’-DDT); toxaphene (Tox-26, 32, 40 + 41, 42a, 44, 50, 62); polychlorinated cyclodienes (endosulfan-I , endosulfan-II, endosulfan-sulphate, heptachlor-exo-epoxide, heptachlor-endo-epoxide, trans-chlordane, cis-chlordane, oxychlordane, chlordene, heptachlor, trans-nonachlor, cis-nonachlor, dieldrin, aldrin, isodrin, endrin) and the individual compounds mirex and trifluralin. In addition, samples were analysed for the polychlorinated biphenyl (PCB) congeners, 18, 28, 31, 33, 37, 47, 52, 66, 74, 77, 81, 99, 101, 105, 114, 118, 122, 123, 126, 128, 138, 141, 149, 153, 156, 157, 167, 169, 170, 180, 183, 187, 189, 194, 206 and 209 (IUPAC numbers) and polychlorinated dibenzo-p-dioxin/furan (PCDD/F) congeners; 2,3,7,8-TCDD, 1,2,3,7,8-PeCDD, 1,2,3,4,7,8-HxCDD, 1,2,3,6,7,8-HxCDD, 1,2,3,7,8,9-HxCDD, 1,2,3,4,6,7,8-HpCDD, OCDD, 2,3,7,8-TCDF, 1,2,3,7,8/1,2,3,4,8-PeCDF, 2,3,4,7,8-PeCDF, 1,2,3,4,7,8/1,2,3,4,7,9-HxCDF, 1,2,3,6,7,8-HxCDF, 1,2,3,7,8,9-HxCDF, 2,3,4,6,7,8-HxCDF, 1,2,3,4,6,7,8-HpCDF, 1,2,3,4,7,8, 9-HpCDF and OCDF.

#### 2.2.5. Sample Preparation and Clean-up

The extraction and clean-up methods for POP have previously been described in full [26]. In brief, dioxin, furan and non-ortho PCB sample extraction and clean-up was performed on a semi-automated 3 column system (first column, Na_2_SO_4_, activated silica and potassium silicate; second column, single use Fluid Management Systems (FMS) silica column; third column, single use FMS activated carbon column). The sample portion containing PCDD/Fs and non-ortho PCBs was eluted from column 3 with toluene, reduced and exchanged to hexane before undergoing further clean-up by sulphuric acid coated silica column followed by potassium hydroxide coated silica column.

Samples for PCB and chlorinated pesticide analysis were extracted on a cold-column and cleaned by gel permeation chromatography, alumina and silica gel columns.

#### 2.2.6. Quantification

The isomer identification and quantification was carried out with HRGC/HRMS using a Hewlett-Packard 5890II (1990–2003) or 6890N (2003–2006) gas chromatograph coupled to an AutoSpec mass spectrometer (Micromass Waters, Manchester, UK). Resolution of mass spectrometer was >10,000 with electron ionization mass spectrometry in the selected ion monitoring mode (GC/EI-HRMS-SIM). Two SIM values were monitored for each isomer group. The added ^13^C-labelled isomers were used as internal standard for each group. Additionally, the recovery rates of the added internal standard compounds were determined.

#### 2.2.7. Quality Assurance

The following quantification conditions were fulfilled for all data presented: (i) the retention time of the native compound was within three seconds of the corresponding ^13^C-labelled isomer; (ii) the isotope ratio of the two monitored masses was within +20% of the theoretical value; (iii) the signal/noise was >3/1 for quantification; (iv) the recovery of the added ^13^C-labelled internal standards was within 40% to 120% and thereby in agreement with EU and US guidelines and official methods; (v) prior to each new series of samples the blank values of the complete clean-up and quantification procedures were determined. Clean-up of samples only commenced when a sufficiently low blank value was obtained. At least once a year the laboratory participates in an international laboratory inter-calibration exercise.

### 2.3. Metrics

#### 2.3.1. Tolerable Daily Intake (TDI)

Various regulatory bodies and food authorities have assessed the levels of chemicals that are safe for human consumption, based upon observed affect levels in animal models. The tolerable daily intake (TDI) refers to a threshold of a chemical which does not appear to carry an appreciable risk. In the current study we have used a variety of sources for our reference TDIs, namely Health Canada and the US EPA, The World Health Organisation and the International Panel on Chemical Safety (IPCS) as well as peer-reviewed literature.

#### 2.3.2. Toxicity Equivalency Factors (TEQs)

Toxicity equivalency factors express the toxicity of similar acting, planar, dioxin, furan and certain PCBs relative to the most potent congener 2,3,7,8-TCDD which is assigned a value of 1.0. The TEQ values applied in the current study refer to Van den Berg *et al.*’s 2005 re-evaluation of TEQ values [28].

## 3. Results and Discussion

### 3.1. Lipid and Fatty Acid Profiles

The majority of categories and brands of seafood oil supplements matched or exceeded manufacturer EPA and DHA specifications, with the exception of three brands which fell slightly below (~10%–30%) the manufacturer specifications (Table 1, Table 2 and Table 3). These related to EPA levels in one enriched fish oil, namely Blackmores Omega liquid fish oil (1700 mg specified *vs.* 1500 mg observed) and DHA levels in Nature’s Way Kidsmart (133 mg specified *vs.* 95 mg observed) and Blackmores Pregnancy and Breastfeeding Gold (125 mg specified *vs.* 85 mg observed). It is noted, that for pure oil capsules it is possible to compensate for EPA and DHA batch variability through marginal capsule volume adjustments. This is however, less readily achievable for formulations, such as the latter two products, and uncontrollable for liquid formulations. A listing of all FA present at >0.5% of the total FA in each product analysed is shown in Table 2.

**Table 2:** A catalogue of fatty acids (FA) quantified, together with the composition (as percent of total FA) of all products. Abbreviations used for oil products are: Swisse Krill Oil (SW); Norkrill (Nor); Bio-organics (Bio-O); Blackmores Omega (B-Ω); Bioceuticals (BCT); Blackmores Joint (B-Joint); Nature’s Way Kidsmart (NWK); Blackmores Pregnancy (B-P); Nature’s Own 1000 mg (NO-1000); Blackmore’s 1000 mg (B-1000); Cenovis 1000 mg (C-1000). Capsules or subsamples from two separate batches of each product were pooled for each FA analysis, except for Blackmores Omega Liquid Fish Oil for which only a single batch number could be sourced. Other:denotes FA present at <0.5% in all products.

Product	SW	Nor	Bio-O	B-Ω	BCT	B-Joint	NWK	B-P	NO-1000	B-1000	C-1000
FA											
14:0	7.3	8	1.9	0.37	4.3	0.12	3.3	5.06	6.3	2.4	6.1
15:0	0.33	0.33	0.15	0.02	0.3	0	0.64	0.46	0.58	0.2	0.44
16:4	0.7	0.77	1.4	0.1	2.6	0.98	0.31	0.24	2.3	1.4	2.3
16:3	0.22	0.23	1	0.05	2.2	0.53	0.27	0.2	1.6	0.9	1.6
16:1ω7c	6.4	5.6	3.3	0.55	5.7	1.07	5.1	4.3	10	4.1	11
16:1ω 5c	0.48	0.54	0.07	0	0.12	0.01	0.14	0.11	0.21	0.07	0.21
16:0	19	23	5	3.2	11	0.18	19	20	17	5.6	15
Br17:1	0.1	0.06	0.16	0.03	0.28	0.12	0.57	0.52	0.35	0.19	0.31
17:1ω 8c + a17:0	0.27	0.25	0.18	0.1	0.3	0.06	0.7	0.6	0.41	0.18	0.41
17:0	0.09	0.1	0.17	0.19	0.28	0.01	0.86	0.83	0.53	0.18	0.47
18:4ω3	2.9	3.4	4.3	1.5	3.6	3.9	1.1	0.89	2.9	3.5	2.7
18:2ω6	2.3	2	1.1	0.75	1.2	0.9	3.4	9.03	1.5	1.1	1.4
18:3ω3	1	1.2	0.68	0.43	0.46	0.59	0.73	1.4	0.81	0.65	0.69
18:1ω9c	11	9.8	5.6	6.5	6.5	1.37	11	13	9	3.9	8.6
18:1ω7c	8.2	7.2	2.3	2.6	2.6	0.94	2.4	2.6	4	2	4.2
18:0	1.01	1.1	1.7	3.6	2.9	0.11	14	7.8	3.6	1.2	3.4
20:4ω6	0.19	0	3.4	0.29	0.5	0.15	1.3	1.3	0.63	0.23	0.41
20:5ω3	20	21	37	35	24	62	6.9	5.7	18	47	19
20:4ω3	0.49	0.52	1.3	1.7	1.1	2	0.48	0.43	0.89	1.6	0.9
20:1ω9c	0.69	0.7	1	3.05	1.4	0.56	1.3	1	1.09	0.73	0.97
20:1ω7c	0.38	0.4	0.26	0.72	0.5	0.07	0.17	0.15	0.45	0.2	0.47
21:5ω3	0.48	0.63	1.3	1.6	1.1	2.4	0.22	0.19	0.68	1.8	0.71
22:5ω6	0.01	0	0.25	0.46	0.17	0.29	0.71	1.1	0.27	0.27	0.22
22:6ω3	12	9.8	20	25	18	16	20	18	11	14	12
22:5ω3	0.46	0.57	2.7	4.8	3.4	4.4	1.3	1.2	2.3	3.7	2.6
22:1ω11c	0	0	0.6	2.3	1.1	0.02	0.67	0.49	0.55	0.2	0.59
22:1ω9c	0.48	0.79	0.15	0.49	0.27	0.05	0.17	0.14	0.2	0.1	0.19
24:1ω9c	0.17	0.19	0.24	0.53	0.53	0	0.37	0.39	0.46	0.15	0.45
Other	3.09	2.39	2.71	4.16	3.61	1.59	3.45	3.39	2.8	1.87	3.19
Total	100	100	100	100	100	100	100	100	100	100	100

Other fatty acids: 14:1ω5c, i15:0, a15:0, 15:1ω6c, 16:2, i16:0, 16:1ω9c, 16:1ω7t, 16:1ω13t, i17:0, 17:1, 18:3ω6, 18:1ω7t, 18:1ω5c, 18:1, 19:1 (2 isomers), 20:3ω6, 20:2ω6, 20:1ω11c, 20:1ω5c, 20:0, 21:0, 22:4ω6, 22:1ω7c, 22:0, 23:0, 24:6ω3, 24:5ω3, 24:1ω11c, 24:1ω7c, 24:0.

**Table 3:** Observed *versus* manufacturer labelled EPA and DHA values of selected fish and krill oil nutriceutical categories and products (labelled and values are rounded to two significant figures). Capsules or subsamples from two separate batches of each product were pooled for each FA analysis, except for Blackmores Omega Liquid Fish Oil for which only a single batch number could be sourced.

Product	Labelled EPA (mg) Per Capsule/Serve	Observed EPA (mg) Per Capsule/Serve	Labelled DHA (mg) Per Capsule/Serve	Observed DHA (mg) Per Capsule/Serve	EPA + DHA (mg) per Max Recommended Daily Serve	Cost (AUD) Per (Labelled) 500 mg DHA + EPA
*Krill Oil*						
Swisse (NKO)	50	55	30	33	240	3.8
Norkrill (Aker Biomarine)	60	86	28	40	180	4.0
*Enriched Fish Oils*						
BioOrganics super liquid	1600	1600	810	860	2400	0.21
Blackmores Omega Liquid	1700	1500	1100	1100	2800	0.24
Bioceuticals omegasure Liquid	1050	1300	750	790	1800	0.28
*Formulations*						
Blackmores Omega Joint	550	590	120	150	2700	0.40
Nature’s way kidsmart	28	33	130	95	320	0.90
Blackmores Pregnancy	25	27	130	86	300	2.7
*18:12 Standard Grade*						
Nature’s Own Odourless 1000	180	180	120	110	2700	0.15
Blackmores Odourless 1000	180	180	120	110	3600	0.10
Cenovis 1000	175	180	70	120	250	0.25

### 3.2. Persistent Organic Pollutants

None of the categories or products analysed in the current study, at their highest recommended dosage, came close to fulfilling tolerable daily intake (TDI) levels for any single analyte (Table 4). Despite the fact that environmental exposure to POPs does not occur to a single residue at a time, but rather to complex and interacting mixtures, this finding is reassuring. As a means of qualitatively comparing and contrasting the eleven products analysed in this study, and providing an overview of chemical summaries obtained, we devised a simple scoring system (Table 5). The five products with the greatest contaminant burden for five key compound groups, plus TEQ values, were ranked from 1–5 with the sample containing the highest concentrations receiving a score of 5. Bioceuticals Omegasure liquid fish oil and Blackmores 1000 mg both carried a cumulative score of 16 reflecting their placement among the top five products for five and four compound/index groups respectively. Blackmores Pregnancy and Breastfeeding Gold formula and Nature’s Own 1000 mg each received a score of 12. Blackmore’s Pregnancy and Breastfeeding Gold formula incorporates tuna oil, sourced from northern hemisphere oceans, thereby likely contributing to the higher contaminant burdens found in this formulation, despite its lower oil content. Blackmore’s Joint formula was the only product which did not feature among the top five products for any analyte or index group.

**Table 4:** Chemical burdens per maximum recommended daily dose (lipid); where relevant, corresponding TEQ (2005) and percent (%) of Tolerable Daily Intake (TDI). Values are presented to two significant figures. Blank squares indicate that values fell below the method level of detection (LOD), which are given as an average concentration for all 11 products (pg/g lipid), whilst grey squares indicate that corresponding congeners were not analysed for that product. Abbreviations used for oil products are: Swisse Krill Oil (SW); Norkrill (Nor); Bio-organics (Bio-O); Blackmores Omega (B-Ω); Bioceuticals (BCT); Blackmores Joint (B-Joint); Nature’s Way Kidsmart (NWK); Blackmores Pregnancy (B-P); Nature’s Own 1000 mg (NO-1000); Blackmore’s 1000mg (B-1000); Cenovis 1000 mg (C-1000). Capsules or subsamples from two separate batches of each product were pooled for each POP analysis, except for Blackmores Omega Liquid Fish Oil for which only a single batch number could be sourced.

Compound (LOD, pg/g Lipid)	TDI ^a^ (pg)/Day Per 60 kg Adult	SW (pg/1 g)	Nor (pg/ 1 g)	Bio-O (pg/4.75 g)	B-Ω (pg/4.75 g)	BCT (pg/4.75 g)	B-Joint (pg/4 g)	NWK (pg/1 g)	B-P (pg/0.86 g)	NO-1000 (pg/9 g)	B-1000 (pg/12 g)	C-1000 (pg/9 g)
*HCH*												
a-HCH (27)				180					260			
b-HCH (32)		72		370					270		690	260
g-HCH (29)		65	33	170					1700			
∑HCH	18,000,000 ^c^	(0.00076)	(0.00018)	(0.004)					(0.012)		(0.0038)	(0.0014)
*DDT*												
o,p’-DDE (46)					480	810.0		1800.0		49,000		
p,p’-DDE (59)					1000	16,000	1200	5000	15,000	120,000	35,000	
o,p’-DDD (37)						840		890	7100	1300		
p,p’-DDD (40)		180		10,000		6700		4900	31,000	20,000	25,000	11,000
o,p’-DDT (43)		260		1300		1100		270	26,000	2000	3600	1000
p,p’-DDT (69)					1400	4400		5100	80,000	140,000	9000	2100
∑DDT	30,000,000 ^c^	(0.0015)		(0.038)	(0.0095)	(0.1)	(0.004)	(0.06)	(0.53)	(1.1)	(0.24)	(0.047)
*Chlordanes*												
trans-Chlordane (60)						4000						
cis-Chlordane (160)						2300						
oxy-Chlordane (1500)					30,000							
cis-Nonachlor (28)						1700		100				
∑Chlordane	30,000,000 ^c^				(0.1)	(0.027)		(0.000028)				
Endosulfan-I (34)	360,000,000 ^c^			390.0 (0.00011)								
*Toxaphene*												
Tox-26 (61)						2400						
Tox-40 + Tox-41 (48)						2300		260	770			3500
Tox-44 (160)						7800		1100				13,000
Tox-50 (23)						6200		330	1100			5200
∑Toxaphene	12,000,000 ^d^					(0.16)		(0.014)	(0.016)			(0.18)
*Chlorobenzenes*												
PeCB (3.1)	60,000,000 ^b^	930 (0.0016)	340 (0.00057)	59 (0.0001)		300 (0.0005)	180 (0.0003)	91 (0.00015)	190 (0.00032)			160 (0.00027)
HCB (2.8)	9,600,000 ^e^	9900 (0.1)	4400 (0.046)	68 (0.00071)		780 (0.0081)	160 (0.0017)	27 (0.00028)	500 (0.0052)			140 (0.0015)
*PCB*		Conc. (TEQ)	Conc. (TEQ)	Conc. (TEQ)	Conc. (TEQ)	Conc. (TEQ)	Conc. (TEQ)	Conc. (TEQ)	Conc. (TEQ)	Conc. (TEQ)	Conc. (TEQ)	Conc. (TEQ)
18 (14)		38	14			150	86		52		140	
28 (10)		48	20		72	370	83		130	590		
31 (10)		45	21		50	230	91		68		190	
33 (10)			13			64	56				150	
47 (4.8)		48	75		210	420	46		240	260	310	
52 (5.4)		130				130	96	13	360			140
66 (4.6)		57	14			850			600	790	600	260
74 (4.0)		30				550			210	280	250	100
77 (0.11)				10 (0.0011)		18 (0.0018)		0.59 (0.000059)	9.4 (0.00094)	54 (0.0054)	110 (0.011)	
81 (0.10)		0.51 (0.00015)			0.48 (0.00014)	0.38 (0.00011)				1.1 (0.00033)		
99 (5.7)		64	12	93		2300		38	820	920	130	510
101 (6.3)		170	42	190		3700	62	69	900	1900	2800	1000
105 (7.6)		34 (0.0010)		130 (0.0039)		1200 (0.036)		19 (0.00057)	210 (0.0063)	1100 (0.033)	1600 (0.048)	720 (0.022)
114 (5.8)						120 (0.0036)					130 (0.065)	
*PCB*		Conc. (TEQ)	Conc. (TEQ)	Conc.(TEQ)	Conc.(TEQ)	Conc. (TEQ)	Conc. (TEQ)	Conc. (TEQ)	Conc. (TEQ)	Conc. (TEQ)	Conc. (TEQ)	Conc. (TEQ)
118 (5.7)		75 (0.0023)	0.017 (0.000001)	340 (0.01)		3500 (0.11)		0.067 (0.000002)	690 (0.021)	2600 (0.078)	4000 (0.12)	1600 (0.048)
122 (6.3)												140
123 (6.3)						55 (0.0017)		13 (0.00039)	110 (0.0033)			230 (0.0069)
126 (0.43)		1.8 (0.18)	0.94 (0.094)	5.5 (0.55)	1.8 (0.18)	4.6 (0.46)				24 (2.4)	50 (5.0)	
128 (8.1)		21		120		1100		69	310	1200	1500	690
138 (6.9)		110	15	790	60	7100	53	380	1800	6700	10,000	4300
141 (4.8)		18		110		850		50	220	1000	1400	640
149 (4.3)		100	19	320		2700	52	120	75	3100	4400	1700
153 (4.1)		140	25	1200	76	9200	75	50	2600	9900	14,000	5500
156 (6.9)						450 (0.014)		30 (0.0009)	150 (0.0045)	530 (0.016)	810 (0.024)	460 (0.014)
157 (5.7)						100 (0.003)				110 (0.0033)	200 (0.006)	91 (0.0027)
167 (5.9)				51 (0.00051)		260 (0.0026)		19 (0.00019)	107 (0.053)	370 (0.037)	550 (0.055)	250 (0.025)
169 (0.28)		1.4 (0.042)	0.86 (0.026)	2.1 (0.063)	0.83 (0.025)					3.6 (0.11)	8.8 (0.26)	
170 (7.7)		18		320	71	1400		150		2100	3500	1700
180 (7.3)				770	130	4000	62	590	2300	6600	9800	4700
183 (5.7)				100	47	630	40	56	190	880	1100	560
187 (6.0)		43		320		1800		190	440	2600	3700	1600
189 (7.7)											250 (0.0075)	
194 (5.3)				110	59	57		83	180	910	1200	640
206 (6.8)								36		200	290	160
209 (2.4)						120		23	106			92
∑PCB (%TDI)	7,800,000 ^b^	1200 (0.015)	270 (0.0035)	5000 (0.064)	1000 (0.013)	4600 (0.59)	800 (0.01)	2500 (0.032)	14,000 (0.17)	45,000 (0.58)	65,000 (0.83)	29,000 (0.37)
*Dioxin/Furans*		Conc. (TEQ)	Conc. (TEQ)	Conc. (TEQ)	Conc. (TEQ)	Conc. (TEQ)	Conc. (TEQ)	Conc. (TEQ)	Conc. (TEQ)	Conc. (TEQ)	Conc. (TEQ)	Conc. (TEQ)
OCDD (0.41)				30 (0.009)	7.4 (0.0022)	73 (0.022)						
2378-TCDF (0.49)		0.54 (0.054)	0.15 (0.015)							0.72 (0.072)		
23478-PeCDF (2.3)			0.19 (0.095)									
123478/123479-HxCDF (0.19)			0.049 (0.0049)									
∑TEQ	120 TEQ/Day	0.23%	0.18%	0.53%	0.17%	0.55%		0.0018%	0.071%	1.5%	4.7%	0.10%

^a^ Values are based on current scientific information and may change; ^b^ Health Canada, 2007 [29]; ^c^ US EPA [30]; ^d^ Man Chan *et al*. (2000) [31]; ^e^ IPCS (1997) [32].

**Table 5:** Ranking (1–5) of products according to analyte or TEQ category where a score of 5 denotes the highest concentration/index value.

	∑HCH	∑DDT	∑Chl	HCB	∑PCB	TEQ	Score
SW	1			5		1	7
Nor				4			4
Bio-O	4					2	6
B-Ω			5				5
BCT		2	4	3	4	3	16
B-Joint				1			1
NWK		1	3				4
B-P	5	4		2	1		12
NO-1000		5			3	4	12
B-1000	3	3			5	5	16
C-1000	2				2		4

Hexachlorocyclohexane (HCH) congeners did not feature prominently in any product profiles, possibly reflecting the slightly lower lipophilicity of this compound group. The DDT group included the highest concentration of any single compound, with 13 ng/g lipid p,p’-DDE detected in Nature’s Own 1000 mg standard fish oil product, equalling a maximum daily dose 120 ng of p,p’-DDE. Notably, only one krill oil formulation (Swisse) showed detectable levels of ∑DDT. p,p’-DDE has repeatedly been found to be one of the dominant congeners accumulating in Antarctic krill and their predators [26,33,34,35,36,37,38]. Previously, the authors have reported a comprehensive overview of baseline contamination in Antarctic krill [26], with HCB and p,p’-DDE dominating the described profiles. Further, team studies on dependent populations of humpback whales (*Megaptera novaeangliae*), found that the profiles of these predators closely mirrored the profiles of their principal prey, Antarctic krill. In the case of the krill oil products analysed in the current study, however, only trace (440 pg/g lipid or daily dose) levels were quantified in the Swisse krill oil brand which may indicate purification through the manufacturing process.

Detectable levels of chlordanes were observed in only three products, namely Bio-Organics Super Liquid fish Oil (30 ng per maximum daily dose), Bioceuticals Omegasure fish oil (4.6 ng per maximum daily dose) and Nature’s Way Kidsmart (0.1 ng per daily dose). Similarly, endosulfan-I was only detected at trace levels (390 pg/g lipid) in BioOrganics Super Liquid fish oil.

Toxaphene structures were not quantified in five of the eleven products due to loss of the analytes during clean-up. However, notable quantities were detected in Cenovis 1000 mg (19 ng/daily dose; 0.15% TDI) and Bioceuticals Omegasure fish oil (16 ng/daily dose; 0.14% TDI). Only trace levels of toxaphene were quantified in Nature’s Way Kidsmart and Blackmores Pregnancy and Breastfeeding formulation. These congeners were undetectable in Blackmores Joint formula and BioOrganics Super Liquid fish oil.

Chlorobenzenes (penta- and hexa-) were quantified in eight of eleven products at levels ranging from 27–9900 pg/maximum daily dose. Antarctic krill products carried the highest levels of chlorobenzene contamination for both penta- and hexa- congeners. The higher levels of particularly HCB, in Antarctic krill oil is not surprising as this has repeatedly been shown to be the compound dominating POP profiles of the Antarctic sea-ice ecosystem food-web [26,33,34]. The finding that levels were greater than any other product categories, sourced from other global regions, however, was unexpected as HCB has been postulated to be approaching global equilibrium [39]. This finding does not support equilibrium conditions and may be reflective of cold trapping or remobilisation processes of the compound in Polar Regions, combined with steady removal from temperate or tropical source regions.

Polychlorinated biphenyls (PCBs) were detected in all products at cumulative levels ranging from 0.01% TDI (Blackmores Joint formula) to 0.94% TDI (Blackmores 1000 mg). Krill oil products were at the lower end of the spectrum (0.034% and 0.015% TDI for Swisse and Norkrill krill oil respectively), as is expected, given the manufacturing applications of these compounds and the lower historical usage in the southern hemisphere.

Dioxins and furans encompass a class of compounds which are not intentionally produced, but originate primarily through the manufacture of other chlorinated chemicals or combustion processes. Whilst the highest detected levels of any single dioxin or furan congener was 73.0 pg/g lipid of octachlorodibenzodioxin (OCDD) found in Bioceuticals Omegasure fish oil, only the krill oil products contained multiple detectable congeners. This is surprising given the low vapour pressure of dioxins and furans which predict long range atmospheric transport in association with particles. This in turn lowers their potential for effective transport to the Antarctic. Toxicity equivalencies (TEQ) are available for dioxins, furans and a sub-set of planar PCBs, and are calculated based upon their common mode of action. The single highest TEQ for any product analysed was obtained for Blackmores 1000 mg standard fish oil product which yielded a TEQ of 5.6 TEQ or 4.7% of the 120 TEQ TDI. Swisse Krill oil, however, also featured among the top five highest ranking TEQ products. Dioxins, furans and planar PCBs are among the POP compounds most effeciently removed by common fish oil cleaning processes [40]. This finding therefore raises two possibilities. Either some of the fish oil products analysed are subject to one or more chemical purification steps during manufacture, reducing their original TEQ values to the ones observed here, with krill oil apparently not being subject to the same procedures. Alternatively processing and handling itself may have introduced contaminants to the krill oil product that were not present in the raw oil. The dioxin/furan profiles of krill oil here do not match the profiles of whole Antarctic krill previously analysed [25], providing support for the latter.

## 4. Conclusions

This study compared a range of readily available fish and krill oil dietary supplements for both their favourable long-chain omega-3 composition and content, as well as their persistent organic pollutant profiles. All products and categories adhered closely to manufacturer specifications and none exceeded chemical guideline thresholds. When krill oil was compared across categories to other fish oil products and formulations, it was the most expensive oil per 500 mg DHA + EPA and adhered to manufacturer EPA and DHA specifications. The two krill oil products were ranked as intermediate in terms of their levels of POP contaminants when compared overall to the remaining omega-3 nutriceutical products selected for this study, with distinct chemical profiles reflecting their geographical region of origin. This study is the first to provide quantitative evaluation of toxicological profiles of Antarctic krill products, an emerging nutriceutical category. It hereby balances consumer information with regard to marketing of krill oil on the basis of product chemical purity. Ongoing monitoring of the pollutant content of fish and krill oil products will become increasingly important as food authorities seek regulatory overview of this rapidly expanding industry.

## References

[1] Psota T.L., Gebauer S.K., Kris-Etherton P. (2006). Dietary omega-3 fatty acid intake and cardiovascular risk. Am. J. Cardiol..

[2] Maclean C.H., Newberry S.J., Mojica W.A., Khanna P., Issa A.M., Suttorp M.J., Lim Y., Traina S.B., Hilton L., Garland R., Morton S.C. (2010). Effects of omega-3 fatty acids on cancer risk. J. Am. Med. Assoc..

[3] Nichols P.D. (2008). Seafood: Best sources of long-chain omega-3 oils and safe consumption. Agrofood.

[4] McCann J., Ames B.N. (2010). Is docosahexaenoic acid, an *n*-3 long-chain polyunsaturated fatty acid, required for development of normal brain function? An overview of evidence from cognitive and behavioural tests in humans and animals. Am. J. Clin. Nutr..

[5] Garg M.L., Wood L.G., Singh H., Moughan P.J. (2006). Means of delivering reccommended levels of long chain *n*-3 polyunsaturated fatty acids in human diets. J. Food Sci..

[6] Deutsch L. (2007). Evaluation of the effect of Neptune Krill Oil on chronic inflammation and arthritic symptoms. J. Am. Coll. Nutr..

[7] Proudman S., Cleland L.G., James M. (2008). Dietary omega-3 fats for treatment of inflammatory joint disease: Efficacy and utility. Rheum. Dis. Clin. North Am..

[8] Peet M., Stokes C. (2005). Omega-3 fatty acids in the treatment of psychiatric disorders. Drugs.

[9] Buhr G., Bales C. (2009). Nutritional supplements for older adults: Review and reccommendations—Part I. J. Nutr. Elder..

[10] Meyer B., Mann N., Lewis J., Milligan G., Sinclair A., Howe P. (2003). Dietary intake and food sources of omega-6 and omega-3 polyunsaturated fatty acids. Lipids.

[11] Holub B.J. (2002). Potential Benefits of Functional Food and Nutraceuticals to Reduce the Risk and Cost of Disease in Canada.

[12] Grant W.B., Cross H.S., Garland C.F., Gorham E.D., Moan J., Peterlik M., Porojnicu A.C., Reichrath J., Zittermann A. (2009). Estimated benefit of increased vitamin D status in reducing the economic burden of disease in western Europe. Prog. Biophys. Mol. Biol..

[13] Nichols P.D., Breivik H. (2007). Fish oil sources. Long-Chain Omega-3 Specialty Oils.

[14] Cansell M., Nacka F., Combe N. (2003). Marine lipid-based liposomes increase *in vivo* FA bioavailability. Lipids.

[15] Bunea R., el Farrah K., Deutch L. (2004). Evaluation of the effects of “Neptune” krill oil on the clinical course of hyperlipidemia. Altern. Med. Rev..

[16] Atkinson A., Siegel V., Pakhomov E., Rothery P. (2004). Long-term decline in krill stock and increase in salps within the Southern Ocean. Nature.

[17] Loeb V., Siegel V., Holm-Hansen O., Hewitt R.P., Fraser W., Trivelpiece W., Trivelpiece S. (1997). Effects of sea-ice extent and krill or salp dominance on the Antarctic food web. Nature.

[18] Food and Agriculture Organization of the United Nations, World Health Organization Report of the Second Session of the Codex Committee on Contaminants in Food. http://www.cclac.org/documentos/CCCF/2008/alinorm/al31_41e.pdf.

[19] United Nations Environment Program (UNEP) (2009). Stockholm Convention on Persistent Organic Pollutants: Ammendments to Annexes A, B & C.

[20] Nicol S. (2006). Krill, currents, and sea ice: *Euphausia superba* and its changing environment. BioScience.

[21] Wania F., Mackay D. (1993). Global fractionation and cold condensation of low volatility organochlorine compounds in polar regions. Ambio.

[22] Alexander J., Froyland L., Hemre G., Jacobsen B., Lund E., Meltzer H., Skare J. (2006). Et Helhetssyn pAa Fisk og Annen Sjomat i Norsk Kosthold (A Holistic View of Seafood in the Norwegian Diet).

[23] Nielsen E., Larsen J.C., Ladefoged O. (2006). Risk Assessment of Contaminant Intake from Traditional Greenland Food Items.

[24] Food Standards Authority Ireland (FSAI) (2002). Investigation on PCDD/PCDFs and Several PCBs in Fish Liver Oil Capsules.

[25] Halliday J. (2006). Dioxins Prompt Second UK Fish Oil Withdrawal. http://www.nutraingredients.com/Regulation/Dioxins-prompt-second-UK-fish-oil-withdrawal.

[26] Bengtson Nash S.M., Poulsen A.H., Kawaguchi S., Vetter W., Schlabach M. (2008). Persistent organohalogen contaminant burdens in Antarctic Krill (*Euphausia superba*) from the eastern Antarctic sector: A Baseline Study. Sci. Total Environ..

[27] Alhazzaa R., Bridle A.R., Nichols P.D. (2011). Replacing dietary fish oil with Echium oil enriched barramundi with C_18_ PUFA rather than long chain PUFA. Aquaculture.

[28] Van den Berg M., Birnbaum L., Denison M., de Vito M., Farland W., Feeley F., Fiedler H., Hakansson H., Hanberg A., Haws L. (2006). The 2005 World Health Organization re-evaluation of human and mammalian toxic equivalency factors for dioxins and dioxin-like compounds. Toxicol. Sci..

[29] Health Canada Federal Contaminated Site Risk Assessment in Canada Part II: Health Canada Toxicological Reference Values (TRVs). http://publications.gc.ca/collections/collection_2012/sc-hc/H128-1-11-638-eng.pdf.

[30] Man Chan H., Yeboah F. (2000). Total toxaphene and specific congeners in fish from the Yukon, Canada. Chemosphere.

[31] International Programme on Chemical Safety (IPCS), World Health Organization Hexachlorobenzene. http://www.inchem.org/documents/ehc/ehc/ehc195.htm.

[32] Bengtson Nash S.M., Waugh C.A., Schlabach M. (2013). Metabolic concentration of Lipid Soluble Organochlorine Burdens in Humpback Whales Through Migration and Fasting. Environ. Sci. Technol..

[33] Waugh C.A., Nichols P.D., Schlabach M., Noad M., Bengtson Nash S.M. (2014). Vertical distribution of lipids, fatty acids and organochlorine contaminants in the blubber of southern hemisphere humpback whales (*Megaptera novaeangliae*). Mar. Environ. Res..

[34] Poulsen A.H., Kawaguchi S., Landrum P., Bengtson Nash S.M. (2013). Dietary exposure of Antarctic Krill to p,p’-DDE; uptake kinetics and toxicological sensitivity in a key polar species. Environ. Pollut..

[35] Poulsen A.H., Kawaguchi S., Kukkonen J.V.K., Leppanen M., Bengtson Nash S.M. (2012). Aqueous uptake and sublethal toxicity of p,p’-DDE in non-feeding larval stages of Antarctic krill (*Euphausia superba*). Environ. Pollut..

[36] Poulsen A.H., Kawaguchi S., Kukkonen J.V.K., Leppanen M., Bengtson Nash S.M. (2011). Altered developmental timing in early life stages of Antarctic krill (*Euphausia superba*) exposed to p,p’-DDE. Sci. Total Environ..

[37] Poulsen A.H., Kawaguchi S., King C., King R.A., Bengtson Nash S.M. (2012). Behavioural sensitivity of a key Southern Ocean species (Antarctic krill, *Euphausia superba*) to p,p’-DDE exposure. Ecotoxicol. Environ. Saf..

[38] Barber J.L., Sweetman A.J., van Wijk D., Jones K.C. (2005). Hexachlorobenzene in the global environment: Emissions, levels, distribution, trends and processes. Sci. Total Environ..

[39] Usydus Z., Szlinder-Richert J., Polak-Juszczak L., Malesa-Ciecwierz M., Dobrzanski Z. (2009). Study on raw fish oil purification from PCDD/F and dl-PCB-industrial tests. Chemosphere.

[40] Maes J., de Meulenauer B., van Heerswynghels P., de Grey W., Eppe G., de Pauw E., Huyghebaert A. (2005). Removal of dioxins and PCB from fish oil by activated carbon and its influence on the nutritional quality of the oil. J. Am. Oil Chem. Assoc..

